# Less Is More: A Case Report on All-on-4 Prosthesis

**DOI:** 10.7759/cureus.54873

**Published:** 2024-02-25

**Authors:** Dhanashree A Minase, Seema Sathe, Anjali Borle, Ankita Pathak, Tanvi Jaiswal

**Affiliations:** 1 Prosthodontics, Sharad Pawar Dental College and Hospital, Datta Meghe Institute of Higher Education and Research, Wardha, IND

**Keywords:** implant-supported fixed prosthesis, full-mouth rehabilitation, atrophied ridges, prosthodontics, prosthesis

## Abstract

Severe alveolar ridge atrophy following tooth removal poses a common and worsening issue in edentulous jaws, affecting patient comfort and quality of life. The All-on-4 approach involves axially positioning two anterior implants and distally orienting two posterior implants, reducing cantilever length and enabling prostheses with 12 teeth. This less invasive option, utilizing distal tilting of implants at 30°, offers biomechanical advantages and has shown short-term success, though long-term research is limited. Immediate-loading treatments are gaining popularity for their high survival rates. Understanding the biomechanics of implant-supported prostheses is crucial for long-term success, emphasizing the importance of optimal occlusal schemes. Challenges such as bone defects and surgical complexity have led to the exploration of achieving full-arch fixed restorations with fewer implants, building upon Brånemark's early work with four implants. The All-on-4 concept, originating in 1999, proves to be a viable treatment option, providing excellent long-term results and improved load distribution in challenging clinical circumstances. This case report explores the successful rehabilitation of a jaw using the All-on-4 implant prosthesis concept, a technique strategically placing four implants in completely edentulous jaws to support a fixed, immediately loaded prosthesis.

## Introduction

Severe alveolar ridge atrophy following tooth removal is a common issue that tends to worsen over time in the edentulous jaw. Various artificial treatment options are available for such scenarios, including complete upper and lower dentures, implant-supported overdentures, and fixed implant-supported prostheses. However, patient satisfaction is notably higher with implant-supported or implant-retained prostheses compared to disposable alternatives [1].

Due to excessive bone resorption and atrophy of the jaws, a mismatch in settlement with removable complete dentures can cause pain for patients, negatively impacting their quality of life [2,3]. The extent of these modifications significantly influences tooth replacement therapy, especially when planning implant-supported restorations [4]. Thorough treatment planning and decision-making become crucial in such cases [5].

In addressing these situations, the All-on-4 concept offers a viable solution. This technique involves the inclination of distal implants in arches where teeth are absent, allowing for the placement of longer implants and greater prosthetic support with a shorter cantilever arm. It also enhances the distance between two implants and improves bone anchorage. The "All-on-4" treatment idea, devised by Paulo Malo, utilizes angled multiunit or straight abutments to provide patients with an immediate, complete jaw restoration using only four implants [6].

This case report details the successful rehabilitation of both jaws with a complete arch prosthesis supported by only four implants, known as the All-on-4 implant prosthesis.

## Case presentation

**Figure 1:**
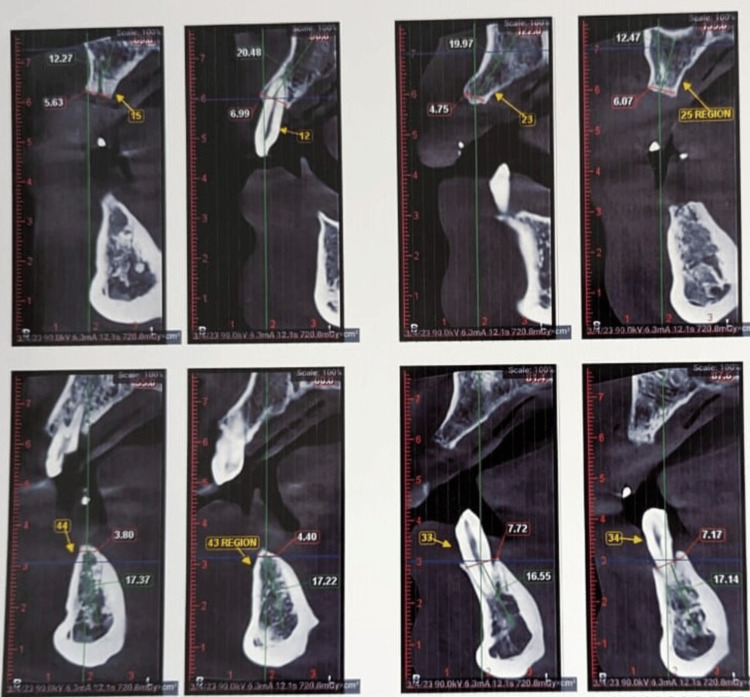
Cone-beam computed tomography in the region where implants were planned in 12, 15, 23, 25, 33, 34, 43, and 44 regions

A 49-year-old male presented to the Department of Prosthodontics with multiple mobile teeth and several missing teeth. Intraoral examination revealed severe, generalized periodontitis with multiple missing teeth, grade III mobility, and recession with remaining teeth. Due to the patient's age, a comprehensive full-mouth rehabilitation plan was devised, involving the placement of four implants in the maxilla and four implants in the mandible, followed by the fabrication of prostheses (Figure 1). 

Implant placement was performed in 12, 15, 23, and 25 regions of sizes 4.5×13 mm, 4.5×13 mm, 4×13mm, and 5×13mm in the maxilla and in 33, 34, 44, and 43 regions of sizes 3.5×13 mm, 4×13 mm, 4×13 mm, and 4.5×13 mm in the mandible. Definitive prosthesis fabrication occurred three months after implant placement, ensuring the establishment of secondary implant stability.

**Figure 2:**
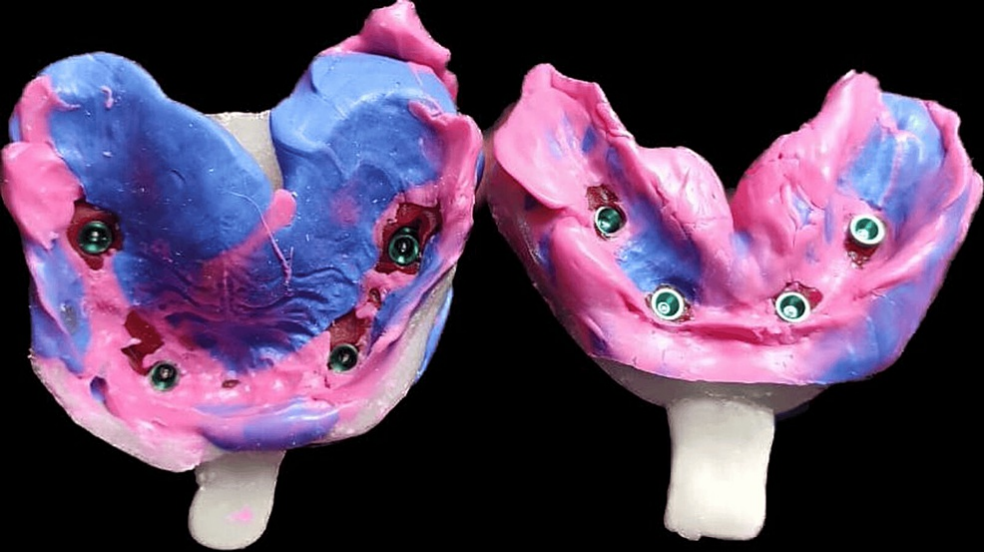
Open tray impression of maxillary and mandibular arches

A preliminary impression using irreversible hydrocolloid alginate (Zhermack Tropicalgin dental alginate (Badia Polesine, Italy)) was made, and a cast was poured with type III dental stone to create a special tray for open tray impressions. Multiunit impression copings on multiunit abutments, attached to the implants for a straight path of insertion, were employed for open tray impressions. The implants were splinted with a wire bar pattern resin (low-shrinkage autopolymerizing resin), and a subsequent open tray impression was made using polysulfide elastomeric impression material (Figure 2).

**Figure 3:**
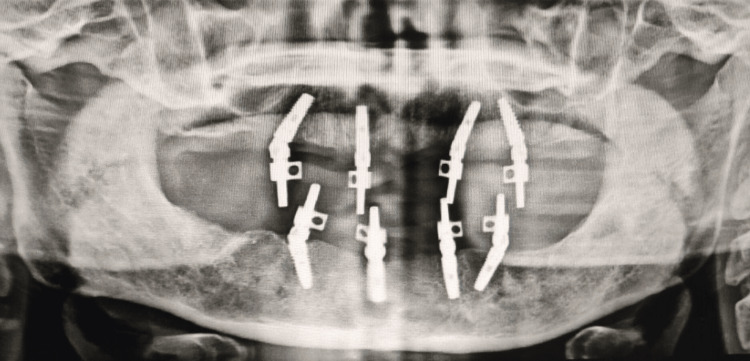
Orthopantamogram

The position of the implants was reassessed using a jig fabricated on the cast. The jig trial was verified with an orthopantomogram for proper fitting of the coping threads and implant (Figure 3).

Jaw relation was recorded, and the inclination of the anterior teeth was determined with vertical and centric jaw relation. The complete assembly was mounted on the semi-adjustable articulator.

**Figure 4:**
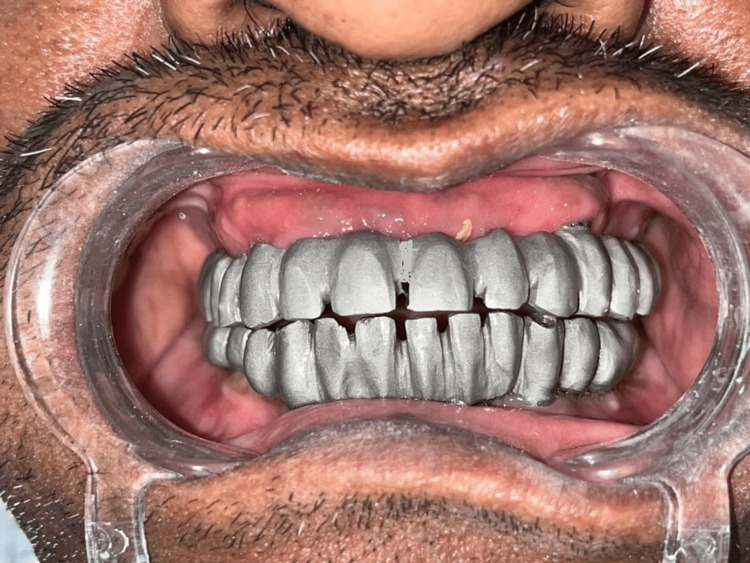
Metal trial intraorally

Following a meticulous evaluation of the inter-ridge distance (19 mm in length), a fixed prosthesis-2 (FP-2) type prosthesis was planned. The cast was scanned with an inEos X5 scanner (Dentsply Sirona, Charlotte, NC) using scan bodies, and the prosthesis was designed digitally. Subsequently, the designed prosthesis was milled using direct metal laser sintering (DMLS). The metal tray was verified intraorally (Figure 4).

**Figure 5:**
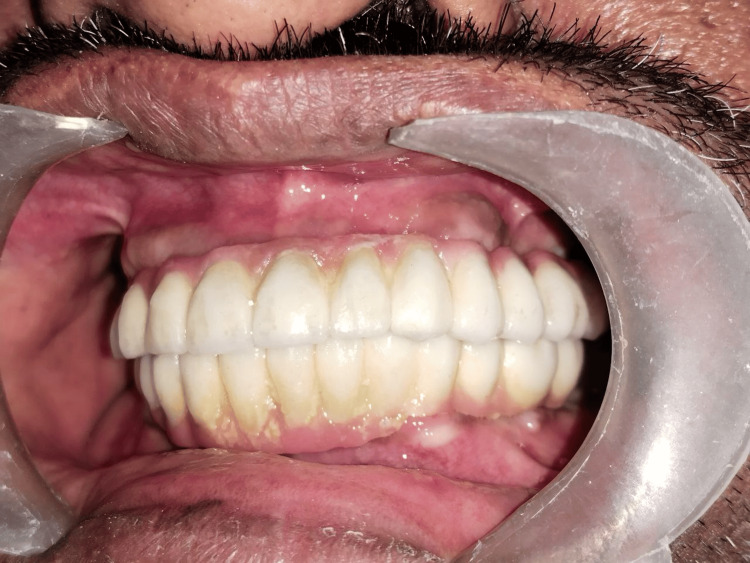
Final prosthesis insertion

An assessment of tooth morphology, size, shape, and arrangement of the interiors was conducted. Shade selection was made according to the patient's facial color. The final prosthesis was inserted (Figure 5).

Adjustments to occlusion were made. Verification of patient aesthetics and phonetics was performed, and the patient was scheduled for regular follow-up appointments. Till now, only a follow-up of one month has been done.

## Discussion

As the distal implants are tilted at 30° to further minimize cantilever length, this less invasive alternative requires fewer implants. Although long-term research is lacking, short-term clinical trials confirm the efficacy of the All-on-4 idea [7,8].

Immediate-loading procedures for edentulous jaws are gaining popularity because of their good survival rates and low occurrence of complications, demonstrating the predictability of implant therapy independent of the loading approach [9]. The primary objective of surgical procedures like angled implant placement or bone regeneration is to restore masticatory function, comfort, and aesthetics, ultimately improving social comfort and self-esteem. Implant-supported fixed prostheses achieve these goals, enhancing patient satisfaction and success rates [10].

Traditionally, dental implants are placed vertically, but challenges arise in fully edentulous jaws, such as poor bone quality, low volume, and the need for bone grafting. Distal tilting of implants proves advantageous by preserving anatomical features, allowing for longer implants with robust cortical anchorage to support prosthetics [11]. This approach offers biomechanical and therapeutic benefits for fixed restorations with less intrusion compared to grafting operations using typical axial implants [12].

The All-on-4 concept originated in 1999, with Mattsson and colleagues treating severely resorbed edentulous maxillae by placing four to six implants in the premaxilla to avoid sinus augmentation [13]. In challenging clinical circumstances involving inadequate bone volume and the need for bone grafting in fully edentulous patients, the use of angled implants in the All-on-4 approach provides a viable treatment option, offering excellent long-term results and better load distribution [14,15].

Understanding the biomechanics of implant-supported prostheses is crucial for long-term success. While natural teeth have a unique feedback system for occlusal awareness, implants differ in biomechanics. The load produced by four implants is found to be higher than that produced by six implants, with no discernible difference between angled and non-angled implants in clinical outcomes [16]. Optimal occlusal schemes are critical for positive outcomes in All-on-4 applications, considering the impact of occlusal loads on implant prosthetic components, similar to other implant therapies. This concept improves cortical anchorage and primary stability, allowing the use of longer implants. The results obtained in various studies show a survival rate for more than 24 months of 99.8% [17].

Various challenges, including bone defects, surgical complexity, economic considerations, and anatomical formations, have led prosthodontists to explore ways to achieve full-arch fixed restorations with fewer implants. Brånemark et al.'s early work laid the foundation for the use of four implants in achieving full-arch fixed prosthetic restorations [18-20].

## Conclusions

The All-on-4 concept emerges as a pragmatic and patient-centric alternative therapeutic option for those with atrophic edentulous jaws. Its affordability, less invasive nature, and expedited treatment timeline make it a compelling choice that not only restores oral function but also enhances the overall well-being of individuals seeking a comprehensive solution to their dental challenges. Fewer implants can create an amazing outcome. By placing only four implants in an arch, complete rehabilitation of the jaw can be done. Hence, we can say that less number of implants can restore the entire arch, less is more, as traditional implant methods often require more implants to support a full set of teeth, which can be time-consuming and complex. Along with that, it reduced surgical intervention by minimizing the amount of surgery required compared to placing a separate implant for each missing tooth. This can lead to a quicker and more comfortable recovery for the patient.
